# Development of Novel Cardiac Indices and Assessment of Factors Affecting Cardiac Activity in a Bivalve Mollusc *Chlamys farreri*

**DOI:** 10.3389/fphys.2019.00293

**Published:** 2019-03-22

**Authors:** Qiang Xing, Lingling Zhang, Yuqiang Li, Xinghai Zhu, Yangping Li, Haobing Guo, Zhenmin Bao, Shi Wang

**Affiliations:** ^1^MOE Key Laboratory of Marine Genetics and Breeding, College of Marine Life Sciences, Ocean University of China, Qingdao, China; ^2^Laboratory for Marine Biology and Biotechnology, Qingdao National Laboratory for Marine Science and Technology, Qingdao, China; ^3^Laboratory for Marine Fisheries Science and Food Production Processes, Qingdao National Laboratory for Marine Science and Technology, Qingdao, China

**Keywords:** scallop, heart rate, heart amplitude, rate-amplitude product, physiological trait

## Abstract

Cardiac activity has been widely used in marine molluscs as an indicator for their physiological status in response to environmental changes, which is, however, largely less studied in scallops. Here, we monitored cardiac performance of Zhikong scallop *Chlamys farreri* using an infrared-based method, and evaluated the effects of several biotic (shell height, total weight, and age) and environmental factors (circadian rhythm and temperature) on scallop heart rate (HR), amplitude (HA), and rate-amplitude product (RAP). Results revealed that size has a significant effect on both HR (negative) and HA (positive), but RAP values are similar in different sized scallops. Age also affects scallop cardiac performance, significantly for HR, but not for HA or RAP. Circadian rhythm affects cardiac activity, with significant elevation of HR, HA and RAP during 1:00–8:00 and 17:00–19:00. With seawater temperature elevation, HR peaks at 30.03 ± 0.23°C, HA at 15.08 ± 0.02°C, and RAP at 15.10 ± 0.19 and 30.12 ± 0.28°C. This suggests HR is a good indicator for thermal limit, whereas HA may indicate optimal growth temperature, and RAP could be an index of myocardial oxygen consumption to indicate myocardium stress. Our study provides basic information on the factors that may affect scallop cardiac performance. It also elucidates the feasibility of HA and RAP as cardiac indices in marine molluscs.

## Introduction

Cardiovascular function conveys important information about whether an organism will survive, and how well they adapt to external environments. As an important parameter of cardiac activity, heart rate (HR) is widely used in vertebrates for evaluating physiological status. For example, HR is an independent predictor of cardiovascular and overall mortality in human (Caetano and Delgado, 2015). In Atlantic cod *Gadus morhua*, HR is also an important indicator for evaluating influences of short-term and prolonged exposure to hypoxia (Petersen and Gamperl, 2010). Similarly in invertebrates, such as Arthropoda (Wilkens et al., 1974; Bamber and Depledge, 1997; Frederich and Pörtner, 2000) and Mollusca (Stenseng and Braby, 2005; Dong and Williams, 2011; Xing et al., 2016), HR is widely applied to study the cardiac responses to various environmental factors, including temperature, salinity, heavy metals, as well as oil contamination. For instance, the changing pattern of HR during temperature challenge has been extensively studied in marine molluscs (Widdows, 1973; Dong and Williams, 2011; Xing et al., 2016), suggesting HR is a good indicator of thermal limits in these animals. Effect of salinity fluctuations on HR of *Hiatella arctica* and *Modiolus modiolus* reveals a significant HR reduction in the initial response to salinity change, and different HR responses during reacclimation depending on the species and salinities (Bakhmet et al., 2012). The impact of heavy metals on organisms can also be investigated by recording the changes of HR. Exposure to copper results in the decay of HR as a function of copper concentration in blue mussel *Mytilus edulis* (Curtis et al., 2000), and bradycardia was observed in limpet *Patella vulgata* exposed to copper and zinc (Marchan et al., 1999). Fluctuations of HR in blue mussels under varying oil product concentrations also reflect the impact of oil contamination on bivalve bioindicators (Bakhmet et al., 2008). All these above indicate that HR is a stable cardiac parameter widely used in vertebrates and invertebrates.

Except for the environmental factors, some biotic factors such as body size, age, and gender also influence cardiac performance. In crustacean *Carcinus maenas*, HR is dependent on body size, with small crabs having a faster HR than the large ones (Ahsanullah and Newell, 1971). Similarly, a significantly negative relationship between HR and shell length was observed in limpet *Patella vulgate* (Santini et al., 2000). In cockroach *Gromphadorhina portentosa*, HR also scales negatively with body size (Streicher et al., 2012). Besides, decline in HR with age was found in human, and gender has a significant effect on HR at age <50 years (Umetani et al., 1998). Consequently, a systematic evaluation on the effects of environmental factors as well as biotic factors on cardiac activity will assist in a better understanding of the physiological status of the organisms investigated.

Based on the in-depth investigation on human electrocardiogram (ECG), there are other parameters besides HR in the ECG which can also provide valuable information on cardiac activity. For example, amplitudes of the P-QRS-T waves represent the variation in membrane potential during the depolarization or repolarization of different pumping chambers. There are also PR interval which reflects conduction through the atrioventricular node, and QT interval that represents the time taken for ventricular depolarization and repolarization. Most importantly, these parameters have been widely used in the clinical diagnosis of various cardiovascular diseases. In contrast, previous studies on cardiac performance in molluscs generally focus on HR, which may provide incomplete information on their cardiac activity.

Bivalves, including clams, oysters, mussels, and scallops, are a large group of molluscs consisting of ∼14,000 species worldwide (Appeltans et al., 2012). They can accumulate different contaminants from ambient water and therefore serve as bioindicators. In addition, some bivalves are of commercial importance, such as Pacific oyster *Crassostrea gigas*, Zhikong scallop *Chlamys farreri*, and Yesso scallop *Patinopecten yessoensis*. Considering their ecological and economic importance, study on the cardiac activity in these animals could benefit ecological surveys as well as aquacultural industry. In this study, we measured the cardiac performance of Zhikong scallop and examined the effects of several biotic factors (shell height, total weight, and age) and environmental factors (temperature and circadian rhythm) on cardiac parameters – heart rate (HR), amplitude (HA), and rate-amplitude product (RAP). It should facilitate a better understanding on the scallops’ physiological status under different conditions.

## Materials and Methods

### Specimens Collection and Acclimation

All Zhikong scallops used in present study were collected from artificial scallop-rearing substrates installed in Xunshan Fishery Group Co., Rongcheng (37°17′18 N, 122°57′56 E).

For the study of size effect, 96 24-month-old healthy scallops with shell height varying from 40.25 to 67.02 mm and total weight ranging from 42.39 to 67.81 g, were collected, respectively. For the study of age effect, scallops with similar shell height (40.53 ± 2.87 mm) were collected for each age group (12-, 18-, and 24-month-old, >=24 specimens for each group) to avoid the interference of size factor. For the study of temperature effect, 24 scallops with similar shell height (48.69 ± 1.91 mm) were collected. For the study of circadian rhythm effect, 48 24-month-old scallops (24 as control group and 24 as treatment group) with similar shell height (52.53 ± 2.62 mm) were collected. To prevent potential genetic effect on the experiments, all individuals were sampled from a pool of various geographical populations including Yantai (37°55′06 N, 120°45′27 E), Weihai (37°17′18 N, 122°57′56 E), and Qingdao (36°06′25 N, 120°32′52 E and 35°57′39 N, 120°16′25 E).

After sample collection, encrusted organisms on scallop shells were removed. The scallops were placed in plastic tanks with filtered and aerated seawater at 15°C for 7 days for acclimation (Bakhmet et al., 2005). To avoid tank effects, all scallops were randomly maintained in several plastic tanks. The water was partially replaced on a daily basis. The animals were maintained without feeding to avoid specific dynamic action. Our experiments were conducted according to the guidelines and regulations established by the Ocean University of China and the local government.

### Cardiac Performance Monitoring

Cardiac performance was monitored by the non-invasive method improved from the technique of Depledge and Andersen (1990). The monitor apparatuses mainly consist of CNY-70 (Newshift, Lisbon, Portugal), AMP 03 amplifier (Newshift, Lisbon, Portugal) and PowerLab 8/35 portable digital recording instrument (ADInstruments, Sydney, NSW, Australia), for signal obtaining, amplifying and recording, respectively. Consecutive cardiac activity waves were recorded using the LabChart software (ADInstruments, Sydney, NSW, Australia).

To detect the effects of size and age on cardiac performance, scallops were maintained at 15°C which is within the optimum temperature range for their growth (14–22°C) (Yu et al., 2010). To investigate the effects of temperature on cardiac performance, scallops were kept in a 5 L beaker immersed in a water bath, allowing the temperature in the beaker to be increased from 5 to 37°C at a rate of 0.2°C per min. To detect the effects of circadian rhythm on cardiac performance, all scallops were kept at 15°C, with the control group being maintained under natural light (a light:dark period of 12 h:12 h), and the experimental group being kept in a darkroom.

### Data Analysis

The heart rate (HR) was counted in beats per min (bpm) (Curtis et al., 2000), and the heart amplitude (HA) representing the peak values of ventricular contraction was calculated in voltage (V). RAP was calculated as HR times HA (bpm^∗^V). For examining the effects of size on cardiac performance, a regression analysis was performed. To detect the effect of age on cardiac performance, one-way ANOVA was conducted followed by Duncan’s test. The HR/HA/RAP increment rate with temperature was obtained for each individual by regression analysis. To compare the differences in HR/HA/RAP between control and experimental groups under circadian rhythm influence, independent sample *t*-tests were performed. Differences and correlations were considered significant when *P* < 0.05. All data were analyzed using SPSS 21.0 (IBM Corp., Armonk, NY, United States).

## Results

### Effects of Size and Age on Cardiac Performance

**FIGURE 1:**
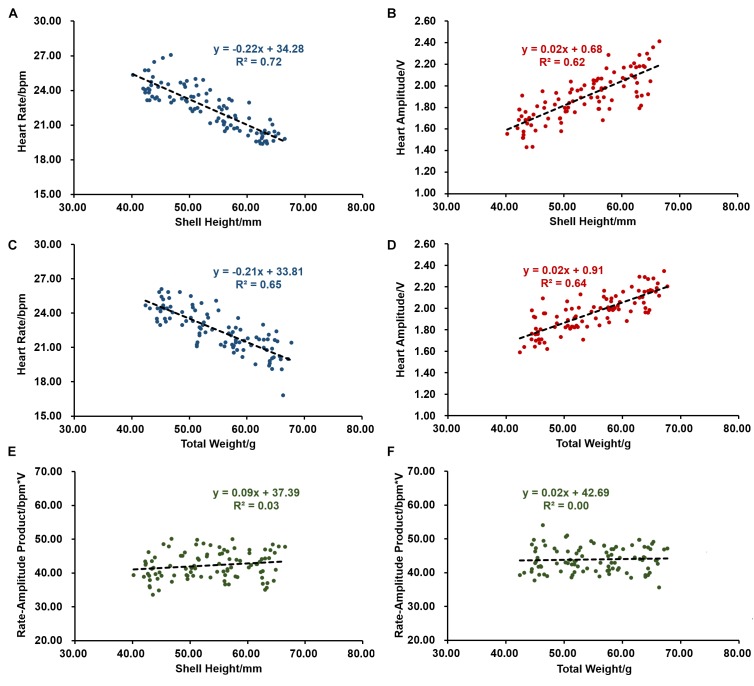
The effects of size on cardiac activity (*N* = 96). **(A)** The negative correlation between heart rate (HR) and shell height. **(B)** The positive correlation between heart amplitude (HA) and shell height. **(C)** The negative correlation between HR and total weight. **(D)** The positive correlation between HA and total weight. **(E)** Correlation between rate-amplitude product (RAP) and shell height. **(F)** Correlation between RAP and total weight.

We first evaluated the effects of size, including shell height and total weight on scallop cardiac performance. As shown in Figure 1A,B, scallops with shell height of 40.25–67.02 mm have an HR ranging between 19.39 and 27.12 bpm, and HA varying from 1.43 to 2.42 V. The average HR and HA are 22.50 bpm and 1.89 V, respectively. Interestingly, shell height decreases with HR (*r* = −0.85, *P* < 0.001), but increases with HA (*r* = 0.79, *P* < 0.001). The effects of total weight on cardiac performance are similar to that of shell height, with the correlation coefficient of −0.80 (*P* < 0.001) and 0.80 (*P* < 0.001) with HR and HA, respectively (Figure 1C,D). The above results suggest that size does affect scallop cardiac performance, and smaller scallops tend to have faster HR but lower HA. Further investigation was conducted on the relationship between size and RAP. As shown in Figure 1E,F, there is no significant correlation between them, indicating that scallops with different shell height and total weight tend to have similar levels of RAP. The average RAP is 42.34 ± 0.41 bpm^∗^V for scallops with shell height ranging from 40.25 to 67.02 mm and 43.91 ± 0.38 bpm^∗^V for scallops with total weight between 42.39 and 67.81 g, respectively.

**FIGURE 2:**
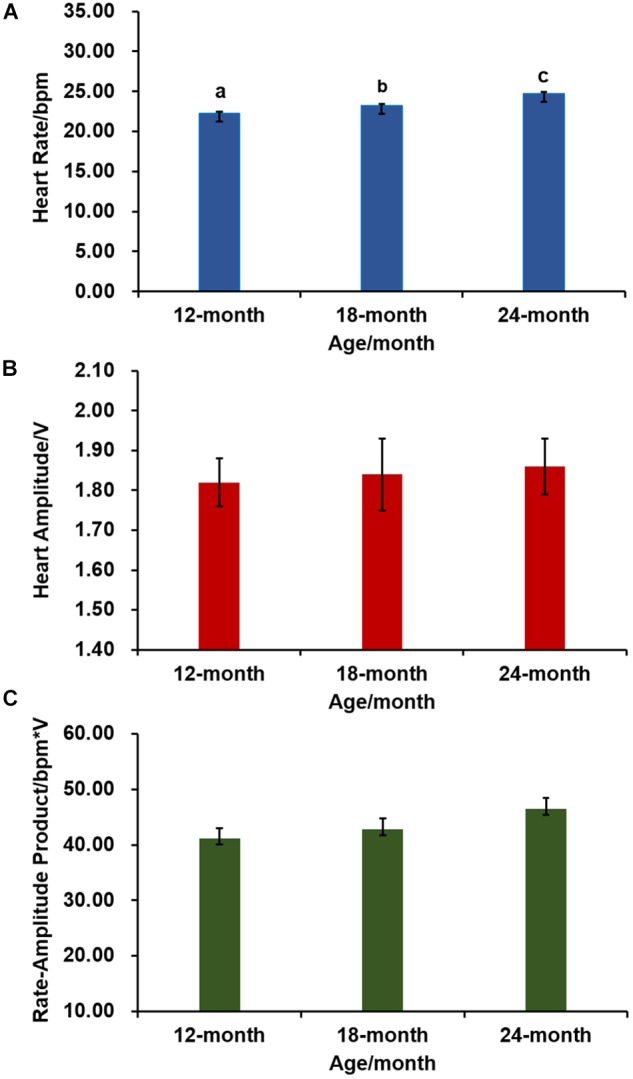
The effect of age on cardiac activity (*N* = 24 for each group). **(A)** HR of Zhikong scallops with different ages. Different letters indicate significant differences (*P* < 0.05). **(B)** HA of Zhikong scallops with different ages. **(C)** RAP of Zhikong scallops with different ages.

We further examined variation of cardiac performance with scallop age. As shown in Figure 2A, HR of 12-, 18-, and 24-month-old Zhikong scallops are 22.08 ± 0.33, 23.11 ± 0.24, and 24.60 ± 0.27 bpm, respectively. The difference among age groups is significant [one-way ANOVA, *F*_(2,69)_ = 20.229, *P* < 0.001], suggesting older scallops have relatively faster HR. But age has no significant effect on HA [one-way ANOVA, *F*_(2,69)_ = 0.061, *P* = 0.941] (Figure 2B) or RAP [one-way ANOVA, *F*_(2,69)_ = 1.986, *P* = 0.145] (Figure 2C).

### Effects of Seawater Temperature on Cardiac Performance

**FIGURE 3:**
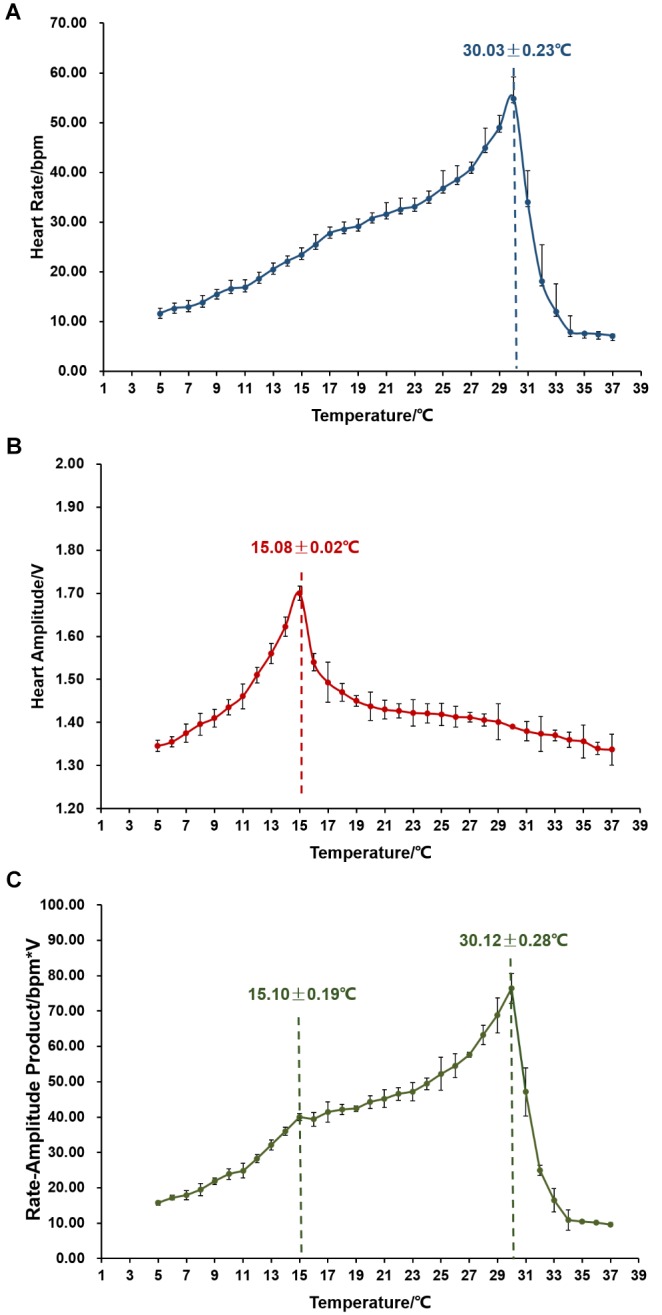
The effect of temperature on cardiac activity (*N* = 24). **(A)** Effects of environmental temperature on scallop HR. The vertical line indicates Arrhenius break temperature (ABT). **(B)** Variations in HA in response to temperature elevation. The vertical line indicates the turning temperature when HA reaches the maximum. **(C)** Changing patterns of RAP with temperature elevation. The vertical line indicates the two peaks in RAP curve.

We then examined the changes in cardiac performance when temperature elevated from 5 to 37°C. As shown in Figure 3A, temperature has a significant effect on scallop HR. HR first increased with the elevation of temperature and the average increasing rate was 1.73 ± 0.04 bpm/°C. After reaching the peak (54.95 ± 4.27 bpm) at 30.03 ± 0.23°C, HR abruptly decreased down to an average of less than 10 bpm at 34°C, with a decreasing rate of 11.73 ± 0.35 bpm/°C. The single peak pattern was also observed in the response of HA to temperature elevation (Figure 3B). According to our data, significantly (*P* < 0.05) higher HA was found from 12 to 16°C, with the maximal HA (1.70 ± 0.02 V) at 15.08 ± 0.02°C. Before the peak, HA increased gradually with an average rate of 0.04 V/°C. Afterward, HA decreased abruptly at the rate of 0.10–17 V/°C, and then declined moderately at 0.01 V/°C until 37°C. Figure 3C showed the changes in RAP with temperature elevation. RAP increased with temperature elevation, reaching the first peak (40.01 ± 1.03 bpm^∗^V) at 15.10 ± 0.19°C with an average increasing rate of 2.42 ± 0.07 bpm^∗^V/°C, and the second peak (76.38 ± 4.27 bpm^∗^V) at 30.12 ± 0.28°C with the rate of 2.42 ± 0.13 bpm^∗^V/°C. Afterward, RAP decreased sharply down to less than 10 bpm^∗^V at 34°C, with the rate of 16.36 ± 0.52 bpm^∗^V/°C.

**FIGURE 4:**
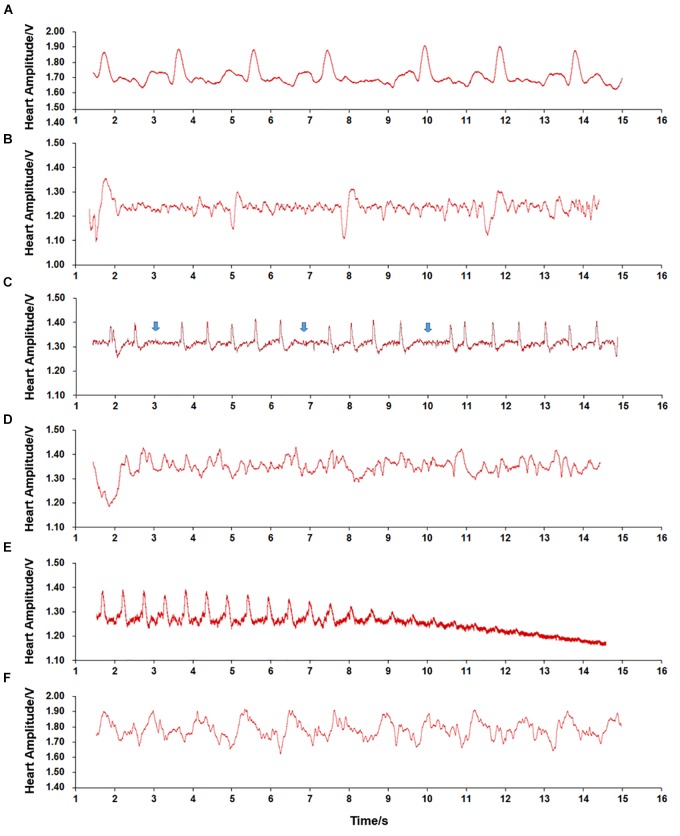
Plethysmograms of cardiac performance recorded during temperature challenge. **(A)** Regular signal pattern recorded in scallops submerged in 15°C seawater. **(B)** Bradycardia recorded in scallops submerged at low temperature (5°C). **(C)** Temporary cardiac arrest recorded in scallops submerged at relatively high temperature (28°C). **(D)** Irregular signal pattern recorded in scallops submerged at high temperature (29°C). **(E)** Cardiac arrest recorded in scallops submerged at 32°C. **(F)** Regular signal pattern recorded in scallops returned to control temperature (15°C).

During temperature elevation, we observed various plethysmogram patterns (Figure 4). In contrast to the normal cardiac plethysmogram at 15°C (Figure 4A), most scallops showed bradycardia with HR as low as 7.06 bpm when submerged at a low temperature (5°C) (Figure 4B). When environmental temperature increased to 28°C, temporary cardiac arrest occurred (Figure 4C, blue arrow). At 29°C, strong fluctuation with irregular signals was observed (Figure 4D). At extremely high temperature (32°C), obvious decrease in HA until disappearance was seen, indicating occurrence of permanent cardiac arrest (Figure 4E). But if scallops promptly returned to the control temperature (15°C), heart beat resumed quickly (Figure 4F). Although the plethysmogram pattern seemed different from the normal pattern (Figure 4A), we assume it represents the recovery of cardiac activity from severe environments. The various plethysmograms recorded at different temperatures indicate that temperature has significant effect on cardiac activity of ectothermic marine bivalve.

### Effect of Circadian Rhythm on Cardiac Performance

**FIGURE 5:**
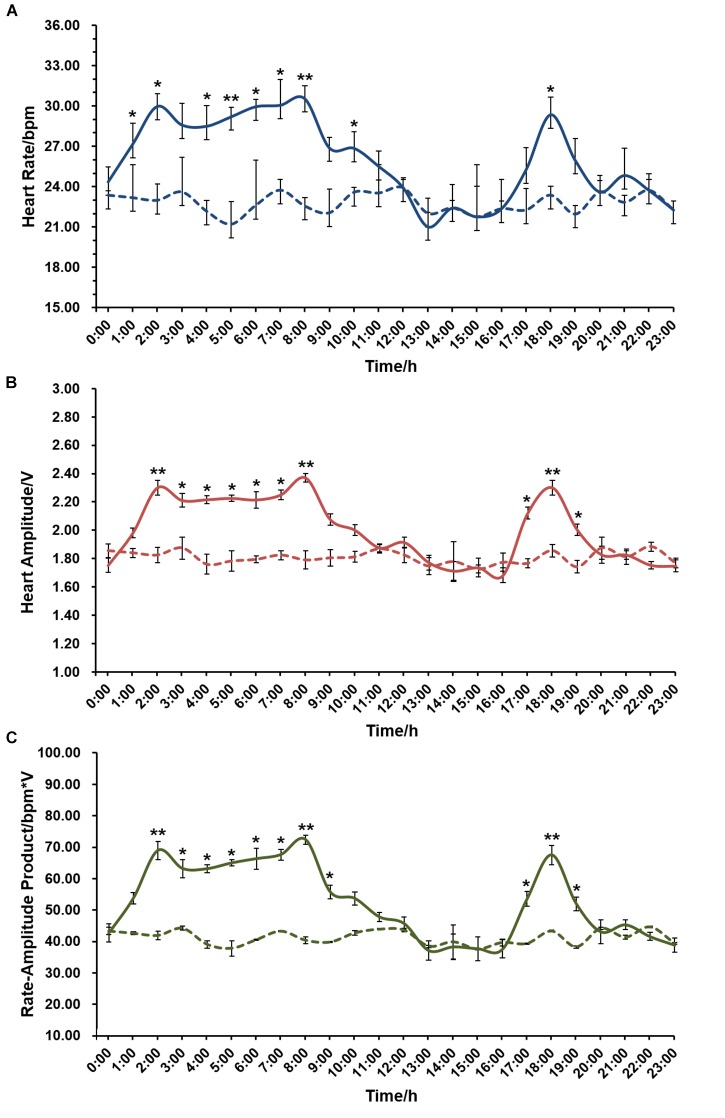
The effect of circadian rhythm on cardiac activity of scallop (*N* = 24 for each group). **(A)** The effect of circadian rhythm on HR. **(B)** The effect of circadian rhythm on HA. **(C)** The effect of circadian rhythm on RAP. The *x*-axis represents a 24 h-period from midnight (0:00) to 11 pm (23:00). The solid and dotted curves indicate HR/HA/RAP monitored under natural light (a light:dark period of 12 h:12 h) and in a darkroom, respectively. ^∗^Indicates *P* < 0.05; ^∗∗^indicates *P* < 0.01.

The effect of circadian rhythm on cardiac performance was also evaluated. As displayed in Figure 5, an average of 22.78 ± 0.27 bpm in HR, 1.81 ± 0.02 V in HA and 41.21 ± 1.66 bpm^∗^V in RAP were recorded in the experimental group that constantly kept in the dark, and there is no significant difference in either HR, HA, or RAP during the 24 h period. In contrast, HR, HA, and RAP showed marked changes when scallops were exposed to natural light. Significantly higher HR was recorded during 1:00–8:00, with HR ranging from 27.16 ± 0.96 to 30.54 ± 0.80 bpm. Correspondingly, we found HA and RAP were significantly higher during this period, with the values ranging from 1.98 ± 0.03 to 2.37 ± 0.03 V, and 53.77 ± 3.52 to 72.37 ± 2.86 bpm^∗^V, respectively. There is another small peak during 17:00–19:00 in HR, HA and RAP curves, with the maximal value of 29.35 ± 1.65 bpm in HR, 2.30 ± 0.05 V in HA, and 67.51 ± 6.14 bpm^∗^V in RAP at 18:00. The above results suggest circadian rhythm has a significant effect on scallop cardiac activity, and HR and HA exhibit coordinate changes under natural light.

## Discussion

Research on cardiac performance has been widely conducted in marine molluscs, such as limpets (Pirro et al., 1999; Santini et al., 2000; Dong and Williams, 2011) mussels (Widdows, 1973; Bakhmet et al., 2005, 2008), oyster (Park et al., 2004) and clams (Taylor, 1976; Bakhmet et al., 2012). It has been demonstrated that mollusc cardiac performance can be affected by many factors, such as body size (Santini et al., 2000), environmental temperature (Widdows, 1973; Santini et al., 2000; Dong and Williams, 2011), salinity (Berger and Kharazova, 1997; Bakhmet et al., 2012), food supply (Widdows, 1973), heavy metals and ammonia (Marchan et al., 1999; Curtis et al., 2000). But current knowledge on marine molluscs’ cardiac responses to environmental factors is based on variations in HR mainly due to two reasons. First, it is widely accepted that HR is a good indicator of physiological status in various animals. Second, HR is relatively stable and easy to calculate. In present study, we developed two cardiac indices – HA and RAP, and evaluated the variation of HR, HA and RAP in response to several biotic and environmental factors. This would contribute to a more comprehensive understanding of scallops’ cardiac activity.

Based on our study, size has significant effects on both HR and HA. There is a significant negative correlation between HR and shell height, as well as total weight. The similar trend of shell height and total weight with HR is expected because previous studies have revealed that these two factors are highly correlated (Du et al., 2016, 2017). Size-dependence of HR was also reported in blue mussel *M. edulis* (Sukhotin et al., 2003) and limpet *P. vulgate* (Santini et al., 2000). It suggests the negative relationship between size and HR may widely exist in various molluscs. Although the effect of size on HA is unknown in other molluscs, scallop HA positively correlates with size. It is noteworthy that RAP remains similar in different sized scallops, suggesting the opposite relationship of HR and HA with size may be a compromise between different sized scallops.

According to our data, age only affects HR but not HA or RAP, with older scallops having significantly faster HR. This is contrary to a report in freshwater mussels, in which obvious decline in HR with age increase was observed (Motley, 1934). Although the molecular mechanism of distinct relationships between HR and age in the two bivalves is unknown, it is an interesting phenomenon worthy of further research.

The independence of RAP with scallop size and age inspires us to consider the possible similarity between scallop RAP and human rate-pressure product (HR^∗^BP, RPP) because RPP is a marker of cardiac function which is relatively stable in healthy people irrespective of height or age (Hui et al., 2000; Overend et al., 2000; Mota et al., 2012). That being said, unlike amplitudes of the P-QRS-T waves in human ECG, HA may represent blood pressure (BP). Moreover, as an index of myocardial oxygen consumption, RPP has strong correlation with the maximal oxygen consumption and is an indicator of myocardium stress (Overend et al., 2000; Mota et al., 2012). This indicates RAP may also be used to measure the workload or oxygen demand of the heart in scallop.

Temperature has been found to be a major environmental factor that affects HR of various marine molluscs (Widdows, 1973; Santini et al., 2000; Dong and Williams, 2011). In Zhikong scallop, temperature has significant effects on HR, HA and RAP. The temperature at which maximal HR reaches – Arrhenius break temperature (ABT), is 30.03 ± 0.23°C, close to the thermal endurance limit of Zhikong scallop we reported before (Xing et al., 2016). Interestingly, a peak in HA was also observed during temperature elevation, which is at 15.08 ± 0.02°C, very close to the optimal growth temperature (15.6–16.0°C) of Zhikong scallop (Wang, 1981). We therefore speculate that recording HA variations during temperature elevation could be an accurate method for assessing an organism’s optimal growth temperature. Similar two peaks were found in RAP curve, one at 15.10 ± 0.19°C and the other at 30.12 ± 0.28°C. This suggests that maximal oxygen consumption may be relatively low when seawater temperature is lower than the optimal growth temperature (1st peak); with the increase of temperature, maximal oxygen consumption increases gradually; cardiac workload reaches the maximum at the organism’s thermal tolerance temperature (2nd peak); afterward, aerobic metabolism decreases due to the inability of the heart to function beyond this critical temperature.

Although the effect of circadian rhythm on mollusc cardiac performance has never been reported before, we found that in scallops, circadian rhythm has a similar effect on HR, HA, and RAP. The same two peaks were found in HR, HA, and RAP curves, with the first one spanning across an 8 h-period from 1:00 to 8:00, and the other across a 3 h-period from 17:00 to 19:00, indicating Zhikong scallops are primarily active at night. This fluctuation pattern is very similar to the daily rhythm of oxygen consumption rate of Zhikong scallop (Zhang et al., 2001) and another bivalve *Coelomactra antiquate* (Meng et al., 2005). Since 8:00 and 18:00 correspond to the natural light shifting, we assume the rhythm of cardiac activity may be related to the changes in light sensed by scallop eyes (Li et al., 2017; Palmer et al., 2017; Wang et al., 2017), and/or the accustomed foraging behavior. From the molecular point of view, origin of biological rhythms consists of clock genes organized in negative and positive feedback loops (Cermakian and Sassonecorsi, 2000; Partch et al., 2014), thus investigation on expression variation of these genes in the daily cycle could help understanding the molecular bases of circadian rhythm of cardiac activity.

To sum up, our study revealed that all the investigated factors including size, age, environmental temperature and circadian rhythm have significant effects on scallop cardiac performance, therefore, these factors should be taken into account in the experimental design. But genetic background could potentially affect cardiac performance (Fujino et al., 1984) and should not be ignored. Our study also suggests that HA could be another informative parameter in the infrared-based cardiac activity measurement. Investigating variation in HA during temperature elevation could be a fast and accurate way for assessing optimal growth temperature of a given mollusc. Moreover, RAP may be an index of myocardial oxygen consumption and could be used to indicate myocardium stress in response to various environmental changes. Considering the variability of cardiac performance among different molluscs, feasibility of HA and RAP as indicators of physiological status in other organisms remains to be studied.

## Ethics Statement

All applicable international, national, and/or institutional guidelines for the care and use of animals were followed.

## Author Contributions

SW, LZ, and ZB conceived and designed the experiments. YaL and HG collected the samples. QX, YuL, and XZ performed the experiments. QX and LZ analyzed the data. QX, LZ, and SW wrote the manuscript. All authors have read and approved the final manuscript.

## Conflict of Interest Statement

The authors declare that the research was conducted in the absence of any commercial or financial relationships that could be construed as a potential conflict of interest.
